# Rising Tides or Rising Stars?: Dynamics of Shared Attention on Twitter during Media Events

**DOI:** 10.1371/journal.pone.0094093

**Published:** 2014-05-22

**Authors:** Yu-Ru Lin, Brian Keegan, Drew Margolin, David Lazer

**Affiliations:** 1 School of Information Sciences, University of Pittsburgh, Pittsburgh, Pennsylvania, United States of America; 2 College of Social Sciences and Humanities, Northeastern University, Boston, Massachusetts, United States of America; 3 Department of Communication, Cornell University, Ithaca, New York, United States of America; 4 College of Computer and Information Science, Northeastern University, Boston, Massachusetts, United States of America; Umeå University, Sweden

## Abstract

“Media events” generate conditions of shared attention as many users simultaneously tune in with the dual screens of broadcast and social media to view and participate. We examine how collective patterns of user behavior under conditions of shared attention are distinct from other “bursts” of activity like breaking news events. Using 290 million tweets from a panel of 193,532 politically active Twitter users, we compare features of their behavior during eight major events during the 2012 U.S. presidential election to examine how patterns of social media use change during these media events compared to “typical” time and whether these changes are attributable to shifts in the behavior of the population as a whole or shifts from particular segments such as elites. Compared to baseline time periods, our findings reveal that media events not only generate large volumes of tweets, but they are also associated with (1) substantial declines in interpersonal communication, (2) more highly concentrated attention by replying to and retweeting particular users, and (3) elite users predominantly benefiting from this attention. These findings empirically demonstrate how bursts of activity on Twitter during media events significantly alter underlying social processes of interpersonal communication and social interaction. Because the behavior of large populations within socio-technical systems can change so dramatically, our findings suggest the need for further research about how social media responses to media events can be used to support collective sensemaking, to promote informed deliberation, and to remain resilient in the face of misinformation.

## Introduction

Social media has transformed the media landscape by providing individuals with the technical capability to compete with mass media in disseminating information, setting agendas, and framing conversations [1], [2]. By providing a technological solution to the costs of directing, aggregating, and disseminating discourse, social media might contribute to political deliberations that are more participatory, support larger exchanges of better information, and promote greater mobilization of political action [3]. Among many uses, tweet streams can both detect breaking news and share information about breaking news events [4]–[6], augment community members' and first responders' awareness of unfolding disasters [7]–[9], and disseminate political speech to both mobilize protests and criticize opposition to established regimes [10]–[12]. Twitter potentially supports more deliberative political participation by facilitating collective sensemaking of fast-paced but ambiguous information, broadening participation in and awareness of policy discussions, and a subjecting messages containing misinformation to greater scrutiny. In many ways, Twitter and other social media offer the promise of advancing collective deliberation toward philosophical ideals in which all individuals are free both to contribute to collective discourse and to directly challenge one another in a public forum [13].

Despite the promise offered by what social media makes technically possible, there is the matter of the socio-technical systems that emerge and evolve around these capabilities. Firstly, there is the question of whether people actually use the affordances of the medium. For example, do Twitter users take advantage of its opportunity for direct public interaction to discuss important issues? Research examining online citizen communication has examined the extent to which messages are deliberative and whether users selectively expose themselves to ideologically polarized messages that insulate them from deliberation [14]–[17]. The openness of social media also makes these discussions vulnerable to misinformation, partisanship, interest groups, activists, and political indifference that undermine offline deliberation [18], [19]. Second, and often overlooked, is the way behavior of and within these systems responds to different conditions and stimuli. Despite growing interest in characterizing the content or behavior on social media [20]–[26], empirical analyses often proceed from the assumption that individual motivations to participate and the behavior of the system are generic and relatively constant [27]. Such an assumption presents communication technologies in an unrealistically deterministic light and disguises the ways that observed changes in social media systems can reveal more general mechanisms of social behavior.

In this paper, we examine how behavior on social media systematically shifts in response to one such condition, the *shared attention to media events*. Our findings reveal that communication behaviors can change significantly under conditions of shared attention both in terms of how individuals produce content and how they attend to it. The primary outcomes of this change are a simultaneous increase in general productivity alongside a general decrease in the diversity of attendance. That is, more people speak, but listening is increasingly focused only on elite speakers. These findings thus suggest that when social media's potential to democratize discourse is technically greatest — when a large number of people are simultaneously connected in virtual space — individual motivations, social norms, and algorithmic prioritization of some tweets over others inhibit its ability to do so.

### Background

Twitter plays an increasingly important role in fostering simultaneous communication around planned events such as political debates [28], sporting events [29], television shows [30], [31], and other large-scale social gatherings [32]. For example, television producers increasingly invite viewers to use a “second screen” so they can “dual screen” and share their reactions to program's content with an audience that is both watching the program and attending to related Twitter streams [33]. Audience members can then see others' responses and interact with each other in real time around their shared interests [34], [35]. Rather than being distractions, the use of textual interactions while attending to video content can bolster the strength of social relationships, even among strangers [36].

Media events can be differentiated from other types of events that encourage dual screening, such as breaking news or viral memes. Unlike breaking news, media events are scheduled in advance and become highly scripted and ceremonial occasions that displace other events and create a collective awareness of its boundaries and content [37]. In particular, media events create conditions in which the audience on social media is simultaneously an audience for traditional media. Interaction is thus about more than the content of the performance or the possibility of social interaction but rather the shared awareness of experiencing the event with others. These broad, collective recognition of media events also potentially undermine the ability for audience members to fragment and selectively expose themselves to polarized media messages [38]–[41].

The simultaneous media use during media events create a social condition we call *shared attention*. We define shared attention as a temporary state in which the individual members of an audience for an event are mutually aware of each other's attention to the event. As we explain below, conditions of shared attention should have important consequences for individuals' dispositions to interact via social media both in terms of how they *produce* and *attend* to messages. In particular, larger potential audiences, altered norms, and high levels of shared understanding can all contribute to shifts in the ways in which users produce content, such as by composing tweets, as well as attend to content by retweeting or replying to the tweets produced by others.

### Our approach

In this study, we use a computational focus group technique [28] to analyze the communicative behavior of Twitter users across varying levels of shared attention during the 2012 U.S. presidential campaign. U.S. presidential campaigns provide natural variations in shared attention. These campaigns generate news and disseminate messages on a daily basis, yet the campaign season includes several planned media events, such as the national party conventions and the candidate debates, that draw national attention both in TV viewership and social media participation [42]. The debates in particular represent high levels of shared attention as they disrupt normal patterns of broadcast television programming and attract large audiences of pundits, partisans, and undecided voters [43]. Social media participation is high during these events with users enthusiastically improvising humorous content [44] and there is some evidence that Twitter use can influence vote choice [45]. Furthermore, the Pew Research Center estimated 11% of the audience for these presidential debates engaged in “dual screening” [46].

Examining how users behave during these media events and comparing it with how they communicate outside of these events permits us to address two research questions. At an individual level, do Twitter users produce and attend to content differently in the context of a media event as compared to their behavior in the context of unexpected news events or normal time? At a collective level, do these individual differences in production and attention alter the collective structure of the conversation during such events?

There are several reasons to expect the shared attention will influence behavior at both the individual and collective levels. Conditions of shared attention are both infrequent and temporary, but this rarity also makes them compelling social experiences that could alter both individual communication behavior as well as the collective structure of audience responses.

#### Concentrated audiences

The potential audience for a given user's content enlarges enormously under conditions of shared attention. Typically, tweets labeled with a hashtag may only be viewed by a few dozen people [47], but under shared attention conditions there may be an audience of thousands that attend to a hashtag for a single topic. However, competition for this larger supply of attention will be more intense. For example, in normal times, tweets on a particular topic are likely to appear on the screens of the small number of people who are paying attention to a particular hashtag at that time, but may stay visible in their feed for several minutes. During a media event about this topic, a tweet would go to a large number of people but be quickly replaced by tweets from others in a matter of seconds.

#### New norms

The uncertain unfolding of the event in real time may also encourage the temporary adoption of new norms regarding the timing of communication [34]. For example, the nature of shared attention may encourage synchronicity in which messages are meant to be immediately read with an understanding that their relevance will quickly fade [48]. Users may also perform other identities or appropriate other affordances of the communication in medium under the conditions of shared attention. For example, Twitter users may shift practices from broadcasting others' tweets to generating their own tweets or engaging in more interpersonal conversations using mentions and replies. Individuals may share particularly informative, funny, or touching messages to communicate to others that they have seen it and are also members in the event [49].

#### Shared understandings

The mutual awareness of the content of the media event can create a common ground for discussion [50]. During media events, individuals can freely construct messages without justifying or explaining their context because they hold common understandings that reduce the need to coordinate content and process [51]. For example, the event relevance of concepts like “Big Bird,” “binders,” and “bayonets” would be difficult to surmise in general, but for viewers of the 2012 U.S. Presidential debates they clearly refer to statements made by the candidates during the media event. Sharing this implicit understanding, individuals can converse without needing to re-explain the context. This common ground may also impose more discipline on the way messages are constructed, as false claims can be easily checked or refuted by a large audience [52], [53].

To examine the extent to which shared attention to media events changes individual and collective communication behaviors, we observed the behavior of approximately 200,000 Twitter users by collecting more than 290 million tweets during eight events, including six media events and two breaking news events, related to U.S. politics that occurred between late August and mid-October 2012. Media events like the national political conventions and presidential debates are compared to a baseline (activity in four normal days preceding each of the four presidential debates) and breaking news events, including the Obama administration's response to the Benghazi attacks and Governor Romney's “47%” statement. We examine the changes in communication patterns, connectivity and concentration, and user responsiveness under the different levels of shared attention imposed by these events.

We focus on two particular behaviors throughout: retweeting and replies. While these practices can be used and interpreted differently across contexts [34], [47], we argue they capture important behaviors in how users signal attention and equality. We treat retweeting as a hierarchical direction of attention, as users draw attention to a message sent by a third user, implicitly asserting the authority of that user for expressing something that others ought to read. By contrast, we view replies as an egalitarian form of production, in which users attempt to alert specific others to their own messages, implicitly asserting an equality of standing where individuals mutually attend to one another's contributions. Because retweeting and replies signal distinct motivations to either allocate followers' attention or demand attention from another user (respectively), each may undergo distinctive changes under the conditions of shared attention to media events.

Our findings indicate that shared attention to media events is associated with two broad effects which we call “rising tides” and “rising stars.” We borrow the term “rising tides” from the aphorism “a rising tide lifts all boats”. In “rising tides” the individual behaviors and collective structure of communication increase, but the distributions of users' production of and attention to information remains similar. It is also possible, however, for average behavior to change due to shifts concentrated in a minority of users. We term this outcome a “rising star” in which the individual behaviors and collective structure of communication shifts in such a way that occurs disproportionately for some users, generally to their benefit. Our results show that the increased attention to social media engendered by media events tends toward the latter effect of “rising stars” by disproportionately concentrating attention to elite users' content.

## Materials and Methods

### Research design

We identified six real world events in which high levels of shared attention were present. Such conditions are difficult to create in the laboratory where it is generally infeasible to enlist or manipulate large scale audiences [54]. Identifying such conditions and appropriate controls is difficult in real-world settings as well. Most media events have relatively unique content. Thus, any effect observed to be correlated with the media event would also likely be correlated with the topic of the event. Without a “control for topic,” inferences attributing association to shared attention would be specious [48].

To assess the impact of this variation in shared attention we identified eight events related to the 2012 U.S. Presidential campaign that occurred over the approximately six-week period of time between late August and mid-October 2012. Six media events were identified during this time: the Republican National Convention (RNC) from August 27 through August 30 (“CONV 1”), the Democratic National Convention (DNC) from September 4 through 6 (“CONV 2”), three debates on October 3 (“DEB 1”), 16 (“DEB 3”), and 22 (“DEB 4”) involving the presidential candidates, and single vice presidential debate on October 11 (“DEB 2”). We contrast these media events with two news events that occurred in the same span of time: the terrorist attack on the American consulate in Benghazi that killed Ambassador J. Christopher Stevens on September 11 (“NEWS 1”) and the release on September 18 of a video in which Mitt Romney argues “47 percent” of Americans are “dependent upon government” (“NEWS 2”). Both of these news events were major stories that dominated media attention for several days.

To provide a baseline, we included activity during the four days before each of the debates when there were no media or news events of similar magnitude (denoted as “PRE”). We term these observation periods “null events.” Although tweet volumes vary regularly throughout the week [55], these null events fell on different days of the week during each of their 96-hour windows reducing the systematic bias of these events. In general, users' behavior during the “typical” time preceding the debate events might have been impacted by the excitements of expected debates and other campaign events, leading to a conservative comparison of changing behavior. This conservative comparison is more appropriate because it ensures that the change we measure is not a result of long-term behavioral drift. Together, these twelve observation periods (four debates, two conventions, two news events, and four “typical” timeframes representing four null events) make up a continuum of varying shared attention: (1) “typical” periods when shared attention is at its baseline level for Twitter as a whole (2) news events that should exhibit low levels of media event-driven behavioral changes since these have diffuse audiences and low mutual awareness of audience members, (3) the national political conventions that should exhibit medium levels of media event-driven changes since partisans selectively expose themselves to the conventions reflecting their political beliefs, and finally (4) the debates that should exhibit the highest levels of media event-driven change as their live and ceremonial nature drive intense shared interest. The array of these observations provides us with natural variation in our independent variable – shared attention.

### Data extraction

Our design requires tracking behavioral change across multiple treatments, thus random sampling from the “garden hose” is inappropriate. We identified a specific sub-population of politically-engaged Twitter users and created a large “computational focus group” [28] to track their collective behavior over time as a panel as follows. If a user tweeted using a hashtag like “#debate” or mentioned one of the candidates' Twitter accounts during any of the four presidential debates and their tweet appeared in the Twitter “garden hose” streaming API [56], the user was selected into our user pool. Next, we collected the complete tweeting history for these users going back to mid-August using Twitter's REST API [57]. Because these queries are expensive owing to rate limits, we prioritized users who tweeted during more of the debates. Thus users who tweeted during all four debates are more likely to be represented in the sample than users who tweeted during only one of the debates. We wrote Python scripts to constantly request the users' past tweets through the “GET statuses/user_timeline” call. Since this method can only return up to 3200 of a user's most recent tweets, over the data collection period (from August to November, 2013), we used parallel processes to request data for each sampled user at least once per week and ensured their tweeting history over the data collection period is complete. The resulting corpus has 290,119,348 tweets from 193,532 unique users including elites such as politicians, journalists, and pundits as well as non-elite partisans and aspiring comedians. Subject to Twitter's Terms of Usage, part of this dataset (the ID numbers for the tweets used in this study) can be shared for replication.

For each of the eight events, we examined tweets made during a 48– to 96–hour window covering the event itself and its aftermath. Within these windows, we examined tweet volumes and identified the hour containing the peak level of cumulative activity. Descriptive statistics for the time of the window, unique users, tweets, retweets, mentions, and hashtags observed in each of the 12 observations (8 events and 4 baseline *null events*) are summarized in Table 1. An “event relevance ratio” is also calculated to validate the differences between events. This ratio is the fraction of tweets during each of the events that containing the names (*e.g.*, “Obama” or “Romney”), candidates' twitter handles (*e.g.*, “barackobama” or “mittromney”), or any of the the events (*e.g.*, “DNC”, “RNC”, “debate”, “benghazi”, “47 percent”, etc.) at the peak time. The event relevance ratio captures the extent to which attention in our observed population is focused on the event topics. The event relevance ratio ranges from 0.08 (PRE) to 0.16 (NEWS), 0.50 (CONV), and to 0.63 (DEB), corroborating our assumption that there is more shared attention to the media events, and to the debates in particular. In the remainder of the paper, we sort these different levels of shared attention into distinct and non-overlapping categories of PRE, NEWS, CONV, or DEB. All tweets within each category's time window is given the same shared attention level label and no tweets have more than one label.

**Table 1:** Summary of datasets.

	PRE	NEWS	CONV	DEB
description	Pre-debate baseline	Benghazi attack, 47% controversy	Republican Nat'l Conv. Democratic Nat'l Conv.	Presidential debates
time	4 days before each debate (20:00–20:00 EDT)	2-day news cycle (14:00–14:00 EDT)	3 days (08:00–14:00 EDT)	4 hours (20:00–02:00 EDT)
duration	96 hours 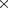 4	48 hours 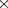 2	66 hours 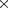 2	6 hours 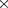 4
peak tweet volume	441,168	131,636	296,138	1,591,513
peak unique users	58,823	30,684	38,864	114,663
event relevance ratio	0.08	0.16	0.50	0.63
shared attention	none	low	medium	high

In Figure S1 in File S1, we provide detailed plots for the distributions of tweet volumes for the hours preceding and following the one-hour window we analyzed.

### Measure of concentration

We measure the level of degree concentration in these Lorenz curves using the Gini coefficient. It is defined as the ratio of the area that lies between the line of equality (the line at 45 degrees) and the Lorenz curve over the total area under the line of equality. The Gini coefficient for a set of users or tweets with degrees 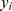 (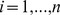) and probability function 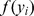 is given by:
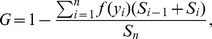
where 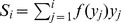 and 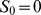. The Gini coefficient is a measure for identifying preferential patterns in general, as opposed to measures such as power-law exponent which can only apply to networks following power-law distribution.

## Results

We analyze the changes in communication patterns across four levels of shared attention: very low (an arbitrary baseline period), low (political news events), medium (national political conventions) and high (presidential debates). First, we compare the differences in activity levels across event types by analyzing differences in individual activity rates at each level of shared attention. Next, we examine the distributions of this activity to understand whether activity differences are broadly adopted by all users or concentrated around a few users. Finally, we analyze the relationship between a user's pre-existing audience size and their position in these activity networks to determine whether skews in the activity distribution are arbitrary or reflect pre-event status.

### Changes in communication activity

**Figure 1:**
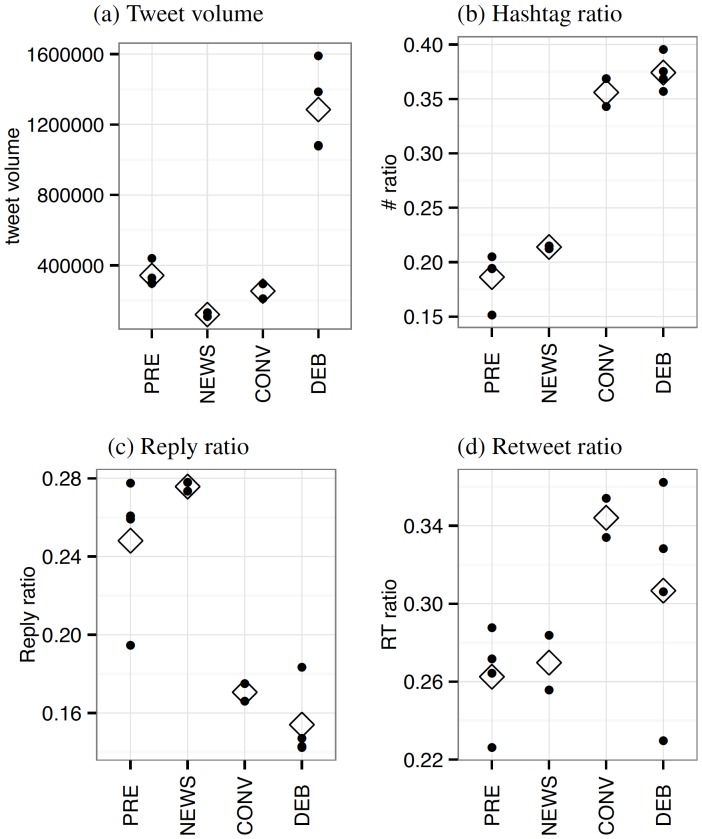
Changes in communication activity. Twitter activity volume change in different events. Diamond shapes indicate the mean value of each category (PRE: pre-debate baseline; NEWS: Benghazi attack and 47% controversy; CONV: Republican and Democratic Natl Conv; DEB: presidential debates). (a) The tweet volumes at the peak hour in the 12 events (including 4 null events). (b) The ratio of tweets with at least one hashtag to the total tweets at the peak hour. (c) The ratio of tweets replying to users to the total tweets at the peak hour. (d) The ratio of retweets to the total tweets at the peak hour. The results show an increase in topical communication (hashtag ratio) and a decrease in interpersonal communication (reply ratio) during the media events over the typical and news events.

Figure 1 plots the changes in communication volumes for each of the four levels of shared attention. Tweet volumes do not appear to vary significantly across the first three levels of shared attention (Figure 1(a)). The tweet volumes for the debates are much larger partly due to our sampling scheme, which focused on those active during the debates (see **Materials and Methods**). The rate of hashtag use nearly doubles during media events over the non-media event rate (Figure 1(b)). Because hashtags are an *ad hoc* way to create a sub-community focused topic by affiliating a tweet with a label [34], [58], the rise of this behavior during media events suggests users are broadcasting diffuse interests in topics.

The fraction of tweets that were replies to one or more users (Figure 1(c)) declines substantially during media events like the debates. This 40% decline in directed communication suggests media events may not only dominate attention, but they also change social media behavior to become less interpersonal and more declarative. At the same time, imitation and re-broadcasting of particular messages appears to increase under shared attention. The ratio of tweets that include any mentions of users in the tweet exhibits similar decline pattern (see Figure S2 in File S1). The retweet ratio during the conventions and debates is substantially greater than under the lower attention conditions, though the mean is greater during the conventions than the debates (Figure 1(d)). Taken together, the results show shared attention is correlated with an increase in topical communication and a decrease in interpersonal communication, suggesting that the shared content of the media event plays a role in organizing the discourse. The increased rate of retweets also suggests that social and psychological processes such as competition for attention or fear of public embarrassment may lead to greater conformity in communication, as individuals are more inclined to repeat what others say than to invent their own messages.

### Changes in distribution

The previous section demonstrated significant changes in the aggregate behavior of the users, however it is unclear whether these differences are driven by broad changes across many users (“rising tides”) or shifts in the activity of a few (“rising stars”). We construct networks of users replying to users (user-to-user) and tweets being retweeted by users (user-to-tweet). Using Lorenz curves, we plot the cumulative distribution of activity in the system for each of the four types of events (see **Supporting Information** for details about activity networks). A Lorenz curve shows for the bottom 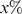 of users or tweets, the percentage 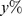 of the activity they generated. More equally-distributed activity is indicated by a linear diagonal while more highly concentrated activity will be more parabolic. A pattern of “rising tides” will be indicated by distributions that are similar to the typical pre-debate events while a pattern of “rising stars” will be indicated by activity during the DEB and CONV events becoming significantly concentrated as compared to the PRE and NEWS events.

**Figure 2:**
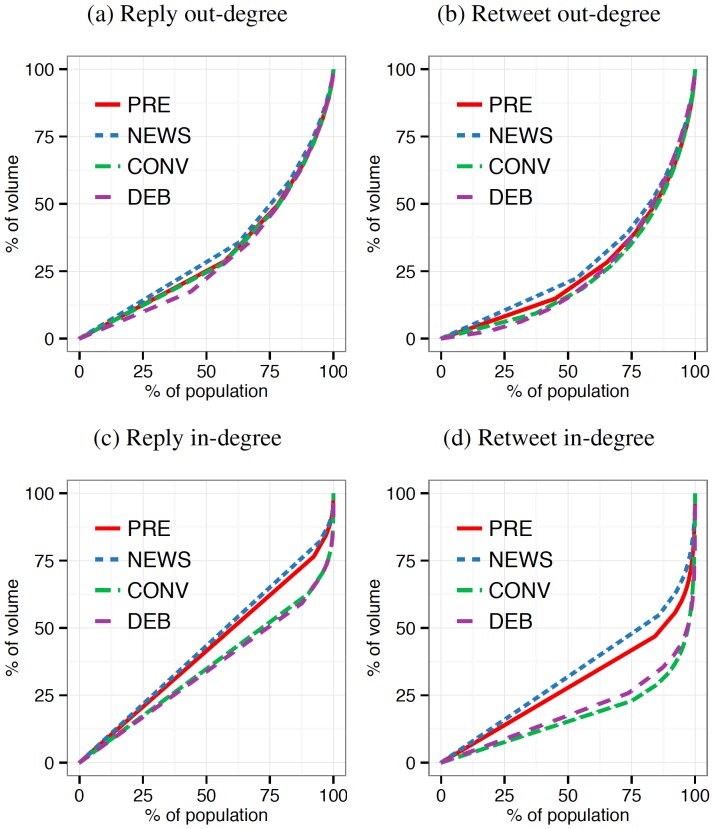
Lorentz curves for cumulative degree distributions of activity. (a,c) The out- and in-degree Lorenz curves for the networks of replies. (b,d) The out- and in-degree Lorenz curves for the networks of retweets (RT). Increasing equality converges toward diagonal line from the origin to the upper-right and increasing inequality converges toward a hyperbola rising to 100% of volume at the 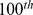 percentile. The out-degrees of activity networks (a,b) show significant similarities across the four event types and comparatively high levels of concentrated activity. The in-degrees show more substantial differences between event types. The convention and debate media events drove increased concentration of reply activity (c) around top users as compared to pre-events and news events. In retweet network (d), the top 25% of users' tweets accounted for approximately 75% of all retweet activity, indicating users' behavior under conditions of shared attention become increasingly concentrated around elites rather than increasingly distributed across many users. (PRE: pre-debate baseline; NEWS: Benghazi attack and 47% controversy; CONV: Republican and Democratic Natl Conv; DEB: presidential debates).

Figure 2 plots the out- and in-degree Lorenz curves for the activity networks of replies and retweets. The out-degree distribution represents individual user level decisions — the kinds of tweets (replies, retweets) each user produced without considering the other users to whom they referred. The out-degree distributions in the activity networks show significant similarities across the four event types. In each case, the level of concentration is fairly high: a few users are responsible for most of the replies to other users (Figure 2(a)) and retweets of users' content (Figure 2(b)). However, the differences in the out-degree distributions between these event types is negligible suggesting that content production follows a pattern of “rising tides” in which concentration remains unaltered. Specifically, the aggregate shifts presented in Figure 1 are the result of changes in behavior by users across the board.

However, the in-degree distribution curves show significant differences between event types. The in-degree distribution represents collective behavior of all other users' activities within our sample directed at a single user or tweet — the kinds of individuals that everyone was paying attention to while paying attention to the event. During periods of low shared attention (baseline and news event), in-degree distributions are fairly flat with only slight leaning toward a very few “stars.” But under conditions of greater shared attention (conventions and debates), the curves bend substantially. Replies are increasingly directed toward a group of approximately 10% of users and away from the bottom 75% of users (Figure 2(c)). We use a two-sample Kolmogorov-Smirnov statistic to test the magnitude and significance of the differences between the baseline and media event distributions. The in-degree distributions for retweet activity (Figure 2(d)) for the conventions and debates show the largest differences from the pre-debate baseline (see Table S1 in File S1 for the statistics of differences). The top 25% of users' tweets account for approximately 75% of all retweet activity under the shared attention conditions, with a much smaller portion being devoted to the other users. In comparison, during the pre-debate and news events, the same amount (75%) of all retweet activity are produced from the top 43% and 50% of users' tweets, respectively. The changes in these distributions for events with higher levels of shared attention suggests that “rising stars” prevail in the attention to content: the concentration of replies and retweets increased despite the increase in retweets and the decrease in replies.

### Rising tides or rising stars?

**Figure 3:**
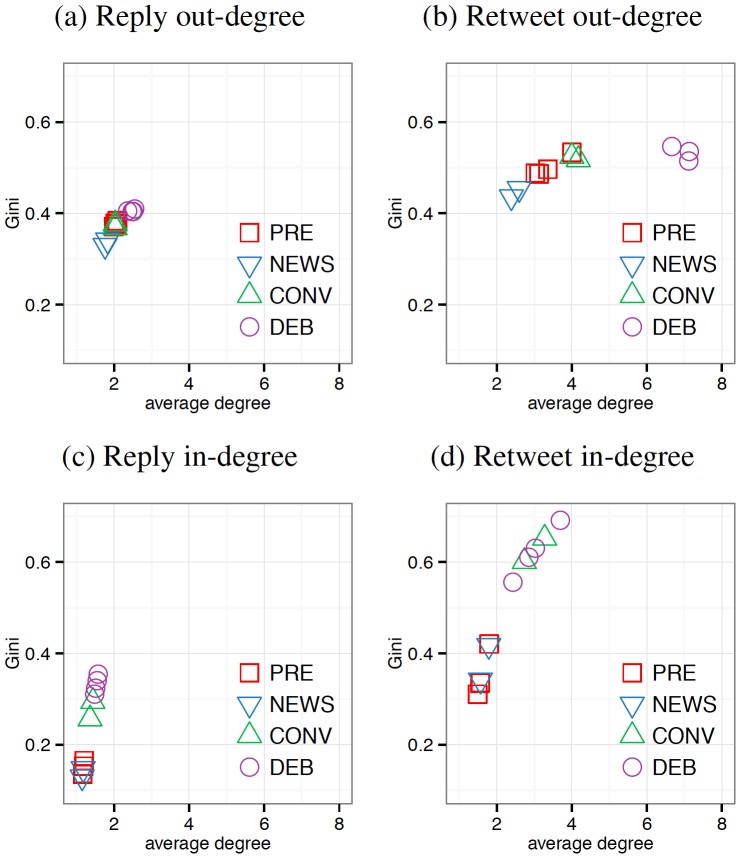
Connectivity-concentration state spaces. (a,c) The out- and in-degree statistics of user-to-user reply network. (b,d) The out- and in-degree statistics of user-to-user retweet network. For each of the twelve observed events, the Gini coefficient (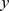-axis) measures the level of concentration of the network's degree distribution, and a lower Gini coefficient indicates a more equal distribution; the average degree of the network (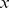-axis) measures average activity of everyone for the event. Across activity types, the in-degrees show consistent patterns of increasing centralization (Gini coefficient) but limited increases in average connectivity degree (average degree) in response to media events while the out-degrees show patterns of increasing degree rather than concentration in response to media events, suggesting that while users across the system become more active during media events, this additional activity predominately benefits a handful of users and tweets. (PRE: pre-debate baseline; NEWS: Benghazi attack and 47% controversy; CONV: Republican and Democratic Natl Conv; DEB: presidential debates).

The preceding analyses suggested the presence of “rising tides” in the production of content as well as the presence of “rising stars” in the attention to content under conditions of shared attention. To compare these effects more directly, we examine the relationship between individuals' connective activity and the concentration of these connections within the networks generated by user activity. Figure 3 plots the average degree of activity in each network against its concentration as measured by the Gini coefficient of its distribution for both replies and retweets (see **Materials and Methods**). Individual-level effects during media events should be reflected in the increased average degree as users increase the extent to which they issue social tweets, increasing the chances that any particular individual is retweeted or replied to and thus increasing connectivity in the graph (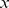-axis). Alternatively, system-level changes during media events should be reflected in the increased Gini coefficient as users concentrate their activity around fewer users or tweets (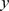-axis).

The phase space can be partitioned into four quadrants: networks in which the users are evenly but poorly connected would cluster around the lower-left, networks with poor connectivity but high levels of centralization would cluster in the upper-left, networks with an even distribution of highly connected nodes would cluster in the lower-right, and networks with highly connected but nevertheless highly concentrated activity would cluster in the upper-right. “Rising tides” will manifest with horizontal movement indicating increases in connectivity without changes in concentration. “Rising stars” will manifest with vertical movement indicating stable connectivity accompanied by an increase in concentration.

As described above, out-degree behavior reflects users' production of tweets. In the user-to-user reply network (Figure 3(a)), the out-degree behavior shows little difference between the events. Though reply rates differ across events (Figure 1), the number of users to whom our sampled users reply appears to increase only slightly for the debates, and the concentration also grows only slightly. In the user-to-user retweet network (Figure 3(b)), the out-degree corresponds to the number of other unique users a user retweets. There is a substantial shift in the out-degree of these networks as the average user retweets between 6–8 individuals during the debates, approximately 4 individuals during the conventions, and less than 4 in the other conditions. This is again evidence of a “rising tide.” Under conditions of shared attention, then, we observe changes in overall activity across users changes (increases in average out-degree) without a substantial change in the concentration of this activity (stable Gini coefficients). Thus, from the median user's perspective, there are more users producing more tweets from more people.

As with Figure 2, the in-degree plots show a very different pattern as users attend to others' tweets. In the user-to-user reply network (Figure 3(c)), the in-degree corresponds to the number of other unique users who reply to a given user. Events characterized by higher levels of shared attention have slighter higher average reply in-degrees, but the concentration approximately doubles from 0.15 to 0.30. This suggests that although the number of users who are replied to on average does not change significantly, the replies that are issued skew heavily toward a few individuals. In the user-to-user retweet network (Figure 3(d)), the in-degree corresponds to the number of unique users retweeting a given user. The in-degree shows a similar pattern for events with high levels of shared attention having more users retweeting them on average (from 2 to 3), but these retweets becoming highly concentrated around specific users.

The connectivity and concentration in other types of activity networks, such as mentions, exhibit similar patterns (see Figures S3 and S4 in File S1). Across these activity types, the out-degrees show consistent patterns of increasing connectivity and limited changes in concentration while the in-degrees show the opposite pattern of marginal growth in connectivity with substantial increases of concentration. In other words, the production of information during media events exhibits patterns of “rising tides,” but the attention to this information by other users leads to “rising stars.” This is not a paradox, but rather a fundamental shift in the nature of the conversation throughout the audience: users of all stripes attend to more users and content than they do typically, but this audience focuses their collection attention on fewer users than is typical. Thus, conditions of shared attention result in a profound homogenization of information intake even as there is greater diversity in what is shared.

### Changes in user responsiveness

The prior sections examined behavioral changes by aggregating all users irrespective of their historical pattern of Twitter use or their position in the Twitter network. These analyses revealed a tendency for Twitter users engaging with media events to participate more actively across the board but to attend more closely to a few users. Yet while this attention is more centered on rising stars, it is unclear who these rising stars are. Are rising stars selected seemingly at random from the tide of users flooding into the system, or are users with existing advantages more likely to seize the benefits of shared attention to media events?

**Figure 4:**
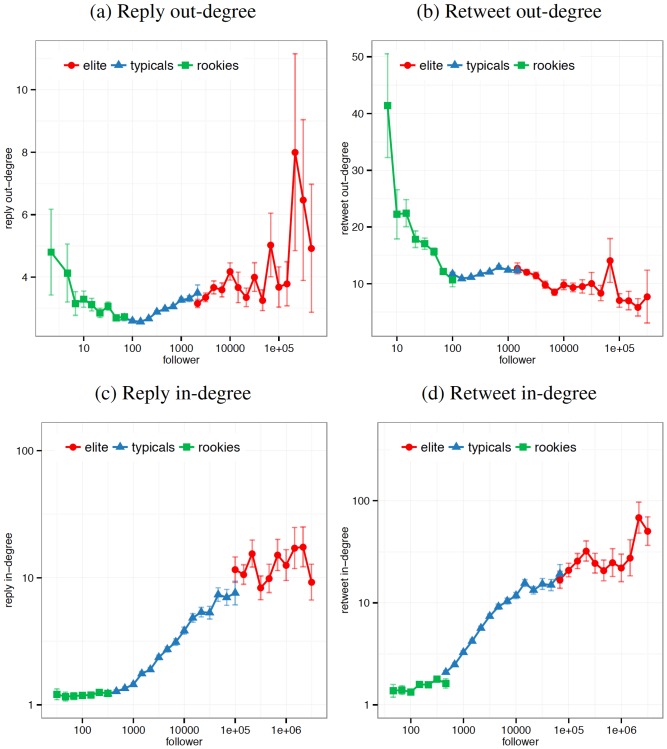
Responsiveness of users during debates. The average increase of the in- and out-degrees for the reply and retweet network during debates compared with the typical events. The 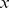-axis are logarithmic bins for all users with 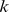 followers and the 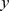-axis measures change of in- or out-degree for all users with 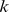 followers. (a) Elites and rookies engage in more interpersonal communication than typicals. (b) Elites retweeted less frequently than other types of users. (c) Elites are largest target of users' replies. (d) Elites have their content retweeted more than other users. In all plots, the 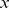-axis plots the number of followers on a log-scale. The 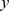-axes are in linear scale in (a,b) and log-scale in (c,d).

We explore the types of users who contribute to and benefit from these shifts in information production and attention. We segment users into three classes based upon their audience size: “elites” are in the 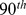 percentile for number of followers (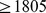), “rookies” are in the 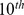 percentile for number of followers (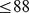), and “typicals” are the middle 80%. Based on this segmentation, Figure 4 plots the distributions for several of the activity types related to the concepts analyzed above, focusing on the average increase of degrees during debates compared with the typical events. We measure the difference between each user's average degree across the four debates and the same user's average degree across the four baseline events.

Although overall levels of interpersonal communication (as measured by replies) decreased in Figure 1, there were significant differences between user classes during the media event. In Figure 4(a), elites and rookies both tended to reply to more users than typical users during the debates. This non-monotonic pattern is interesting as it suggests normative and strategic dimension for interpersonal communication during media events. Rookies may fail to realize that most users (the typicals) are not attending to interpersonal relationships during media events and vainly attempt to engage them in conversation. On the other hand, elites may use these events to cultivate strategic relationships by engaging other elites they know to be active and engaged as well as performing for the rest of their audience. In Figure 4(b), rookies show a significantly higher frequency of retweeting content while elites rarely retweet content. The difference in these propensities is revealing as it suggests highly strategic behavior on the part of elites being selective in whom to award attention.

While there is interesting behavioral variation on the production-side of the shared attention equation, the story of who benefits from this attention is unambiguous: elites. In Figure 4(c), elites are much more likely to be replied to by other users while rookies are effectively ignored relative to their usual rate for garnering attention. In Figure 4(d), elites are much more likely to be retweeted while rookies' content, as before, is eschewed during this period of heightened attention.

As shown in Table 2, for example, the most re-tweeted user during each of the first three debates (first two presidential plus vice-presidential) was comedian/pundit Bill Maher. Barack Obama himself was also in the top 5 during these debates, which also featured conservative commentator Sean Hannity and conservative comedian Dennis Miller. Despite the potential for conditions of shared attention to provide a coherent and egalitarian space for discussion and creativity, users with largest audiences nevertheless become the focus of attention. Furthermore, these tweets have substantial variation in their deliberative intent with many containing irreverent and humorous content rather than assertions intended to motivate political action or re-evaluate policy preferences. These findings provide additional evidence that the dynamic of information production and attention for media events within this population is better described by “rising stars” than “rising tides”.

**Table 2:** Most retweeted users and their tweets across four debates.

Event	Most re-tweeted tweets
DEB 1	@billmaher: “Its Obama's anniversary - he's got to deliver twice tonight!”
	@BlGBlRD: “WTF Mitt Romney…:(”
	@BarackObama: “Watch live: President Obama discusses his specific plans to keep us moving forward in the first presidential debate. http://t.co/3JJ2Yhlt”
	@DennisDMZ: “Jim Lehrer…be a mensch and get out of the way…These are big boys, you are Snooki.”
	@Obama2012: “Jim Lehrer: "What are the major differences between the two of you about how you would go about creating new jobs?"”
DEB 2	@billmaher: “Debate must be about to start, Chris Mathews breathing into paper bag”
	@BarackObama: “Watch live: @JoeBiden lays out the Obama-Biden plan to keep us moving forward. http://t.co/tK4y3oZR #ReadyForJoe”
	@seanhannity: “Biden is going to be "Talking Point Joe" all night #VPDebate”
	@KarlRove: “Ala 2004, are those packs on Raddatz's back a way for ABC higher ups to feed her questions? Just kidding. #debate”
	@TruthTeam2012: “The President is determined to find those responsible for the attack in Libya and to bring them to justice.”
DEB 3	@billmaher: “100 people around stage - Mitt sees more than that at his breakfast table”
	@BarackObama: “Watch live: President Obama lays out his specific plan to keep growing the economy in tonight's presidential debate. http://t.co/BsVgAWvQ”
	@seanhannity: “Middle class crushed last 4 years… #PresidentialDebate2012”
	@TruthTeam2012: “Romneys 12 million jobs claim? 4 Pinocchios: http://t.co/uR4eLIek”
	@DickMorrisTweet: “#debates and there is nothing else holding Obama up. So all Mitt needs to do is be good as he was in the last debate. Obama's performance”
DEB 4	@YABOYMITT: “GAME TIME BITCHES! THEY SEE ME ROLLLLIIINNN THEY HATTTIINNN #YABOYMITT”
	@tyleroakley: “Watching and live-tweeting throughout the Presidential #Debates: http://t.co/rOtOOU8u RT if you're watching with me!:]”
	@realjohngreen: “(I'll mostly be retweeting other people's debate jokes, but occasionally I will sound my barbaric yawp over the rooftops, etc.)”
	@DemetriMartin: “I am live tweeting the debate. This tweet is about it but not directly.”
	@GlobalGrind: “President Obama's leadership has made America stronger, safer, and more secure than we were 4 years ago. #StrongerWithObama”

These findings are consistent with prior research [27], but in light of our previous findings suggest novel dynamics within the collective conversation. First, the variations in behavior across users of different pre-existing status suggests that typicals and elites are aware of the dynamics of shared attention in a manner that rookies are not. Comedian Demetri Martin, the 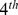 most retweeted user during the final debate, appropriately stated “I am live tweeting the debate. This tweet is about it but not directly.” Martin's tweet highlights both how conditions of shared attention alter norms for information dissemination as well as his privileged position as a media personality with a large audience. Examining Figure 4 column-wise, rookies reach out interpersonally via replies but do not receive proportionate responses. By contrast, typical users appear to “know their place” as they appear to recognize that attention is scarce and others are unlikely to respond. Interestingly, elites actually increase their rate of reply, perhaps in an attempt to initiate dialogue with other elites or in awareness that they have moved to the center of attention. Elites also appear to guard their status, indicated by their restraint in retweeting others at a time when both rookies and typicals increase retweeting behavior, suggesting a reluctance to “anoint” others as worthy of attention through retweeting their content. The rising tide of retweets is supplied by the other users, in particular, rookies. Here rookie retweets may be used as safe ways to express ideas by using the words of others to stand in for their own voices, a phenomenon that might be expected given the intensity of shared attention from a large audience. These dynamics suggest that under shared attention, conversations self-organize into a contemporary two-step flow [59], reminiscent of the format used in broadcast media where elites are appointed to have a conversation among themselves while others receive their wisdom by watching from home [60].

## Discussion

Previous work examining the dynamics of socio-technical systems like Twitter relied upon the assumption that the behavior of users within these systems are self-similar and stable across changing social contexts. Our findings however complicate this assumption by demonstrating large populations of users change their individual and collective patterns of producing and attending to information under conditions of shared attention to media events. At the individual user level, information sharing behaviors, like using hashtags or retweeting, increased during media events, while interpersonal communication behaviors, like replies, decreased. This lends support to the idea that the condition of shared attention created by media events serves to make individuals more group focused and less involved with their normal social foci.

At the collective level, we examined whether these media events created “rising tides” that changed the behavior similarly across the system or if these events created “rising stars” that reinforced the attention and audience for already elite users. While there were increases in both the overall production of and attention to content by users in our population, shared attention clearly rewarded some users over others. References to users or tweets through retweets or replies became significantly more centralized during media events without correspondingly large changes in the average behavior of users. Crucially, the beneficiaries of this newfound attention were not distributed throughout users with different numbers of followers, but concentrated among users with the largest pre-existing audiences.

Despite the potential for social media to create larger public squares with more diverse voices speaking, occasions for large-scale shared attention such as media events appear to undermine this deliberative potential by replacing existing interpersonal social dynamics with increased collective attention to existing “stars”. The particular socio-technical mechanisms that drive the behavioral changes we have identified remain unclear. On one hand, temporary social norms of exuberant information sharing and psychological processes of sensemaking may be the primary factors in these individual and collective behavioral changes. For example, the uncertainty of live events may predispose users to seek information from authorities and their expert sensemaking processes rather than from their peers. On the other hand, the algorithmic infrastructure of Twitter's technical systems could also privilege certain tweets and practices. For example, Twitter announced in September 2013 that it would allow “verified” accounts (users whose identities have been declared to be authentic by Twitter) to filter replies, mentions and, retweets to only include messages and notifications from other verified accounts [61]. Although our analysis pre-dates the implementation of this feature, it nevertheless points to both the demand from elite users to manage the connections they attend to as well as the technical capability for Twitter to privilege some users' messages over others.

These behavioral changes during shared attention to media events also have implications for ensuring the resilience of socio-technical systems for political communication in the face of misinformation. The engaging nature of these events can potentially make audience members less critical of incoming information as well as complicate the ability for users to establish the credibility of tweets and their authors [62]–[64]. Combined with our findings about concentrated attention to elite voices and diminished use of interpersonal communication, these factors could combine to create ideal conditions for rumor persistence, belief polarization, and the dissemination of misinformation that can (intentionally or unintentionally) undermine deliberation. However, the attention given to elite users during media events may provide opportunities for good-faith actors to limit the spread of misinformation by using content-based strategies of issuing repeated retractions, emphasizing facts instead of repeating myths, giving pre-exposure warnings about the likelihood of future information, offering simple rebuttals to complex myths, and fostering norms of strong skepticism [65].

Our analyses have several limitations that are opportunities for future work. Our data included only eight major events across a relatively brief six-week period of time on topics related to politics, limiting the generalizability of these findings to other domains. Future work might explore whether similar patterns are found in other types of media events such as sports (*e.g.*, Super Bowl) and awards ceremonies (*e.g.*, Academy Awards) or across longer spans of time such as an entire political campaign. Despite the size of user cohort whose behavior we analyzed and our intent to capture the behavior of politically-engaged users, the sampling strategy we employed potentially oversampled active users during the debates. Alternative sampling strategies might uncover weaker or different social dynamics. A variety of more advanced metrics and features such as waiting times between tweets and assortative degree mixing could be used to analyze social dynamics of elite users attending to other elites' content. The content and motivation of these tweets was also not analyzed for sentiment, discursive intent, or user background that could be revealed by participant interviews, topic modeling, or content analysis.

By considering not only changes in the overall level of activity, but changes in the structure of the networks of users and tweets, we identified the influence of several processes operating at micro- and macro-levels. Our findings demonstrate that changes in the aggregate levels of activity during media events are driven more by “rising stars” as elite users become the focus of collective attention rather than being driven by “rising tides” as users distribute their attention more broadly to new and diverse voices. Social media like Twitter are not only sites for political communication among politicians and their supporters, they are increasingly becoming spaces for otherwise segmented audiences to come together in a third space to participate in consequential events.

## Supporting Information

File S1
**Supporting figures and table.** Figure S1, Tweet volume per minute. Number of tweets per minute in the 12 datasets. (a–d) The six hours during the four debate events (“DEB”). For other categories, we plot the six hour volume centering around the peak within the data range: (e–h) Normal period prior to the debate evenings (“PRE”). (i,j) National convention events including RNC and DNC (“CONV”). (k,l) Breaking political news events including Benghazi attack and Romney's 47-percent video (“NEWS”). Figure S2, Changes in communication volume. Diamond shapes indicate the mean value of each category. This figure shows the ratio of tweets mentioning a user to the total tweets at the peak hour. Figure S3, Lorentz curves for cumulative degree distributions of activity. Increasing equality converges toward diagonal line from the origin to the upper-right and increasing inequality converges toward a hyperbola rising to 100% of volume at the 100th percentile. Figure S4, Connectivity-concentration state spaces. For each of the twelve observed events, the Gini coefficient for the network's degree distribution is plotted on the 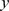-axis and the average degree of the network is plotted on the 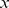-axis. Table S1, Kolmogorov-Smirnov test (K-S test) for comparing the PRE curves with the remaining three curves in other conditions.(PDF)
