# Supplementary material for: Rising Tides or Rising Stars?: Dynamics of Shared Attention on Twitter during Media Events
Source: PLoS One. 2014 May 22;9(5):e94093. doi: 10.1371/journal.pone.0094093 (PMC4031071; doi:10.1371/journal.pone.0094093)
Supplement: File S1 — Supporting figures and table. Figure S1, Tweet volume per minute. Number of tweets per minute in the 12 datasets. (a–d) The six hours during the four debate events (“DEB”). For other categories, we plot the six hour volume centering around the peak within the data range: (e–h) Normal period prior to the debate evenings (“PRE”). (i,j) National convention events including RNC and DNC (“CONV”). (k,l) Breaking political news events including Benghazi attack and Romney's 47-percent video (“NEWS”). Figure S2, Changes in communication volume. Diamond shapes indicate the mean value of each category. This figure shows the ratio of tweets mentioning a user to the total tweets at the peak hour. Figure S3, Lorentz curves for cumulative degree distributions of activity. Increasing equality converges toward diagonal line from the origin to the upper-right and increasing inequality converges toward a hyperbola rising to 100% of volume at the 100th percentile. Figure S4, Connectivity-concentration state spaces. For each of the twelve observed events, the Gini coefficient for the network's degree distribution is plotted on the -axis and the average degree of the network is plotted on the -axis. Table S1, Kolmogorov-Smirnov test (K-S test) for comparing the PRE curves with the remaining three curves in other conditions. (PDF) [file pone.0094093.s001.pdf]

Supporting Information

Rising tides or rising stars?: Dynamics of shared attention on Twitter during media events
by Yu-Ru Lin, Brian Keegan, Drew Margolin and David Lazer

Table of Contents

S1 Dataset detailed figures **1**

S2 Features of communication **2**

S3 Patterns of other activity networks **3**
S3.1 Changes in communication (mention) 3
S3.2 Changes in distribution 3
S3.3 Changes in concentration 4

S4 Statistics of changes in distribution **5**

References **7**

List of Figures

S1 **Tweet volume per minute.** Number of tweets per minute in the 12 datasets. (a-d) The six hours during the four debate events ("DEB"). For other categories, we plot the six hour volume centering around the peak within the data range: (e-h) Normal period prior to the debate evenings ("PRE"). (i,j) National convention events including RNC and DNC ("CONV"). (k,l) Breaking political news events including Benghazi attack and Romney's 47-percent video ("NEWS"). 2
S2 **Changes in communication volume.** Diamond shapes indicate the mean value of each category. This figure shows the ratio of tweets mentioning a user to the total tweets at the peak hour. 3
S3 **Lorentz curves for cumulative degree distributions of activity.** Increasing equality converges toward diagonal line from the origin to the upper-right and increasing inequality converges toward a hyperbola rising to 100% of volume at the 100th percentile. 4
S4 **Connectivity-concentration state spaces.** For each of the twelve observed events, the Gini coefficient for the network's degree distribution is plotted on the y-axis and the average degree of the network is plotted on the x-axis. 5

List of Tables

S1 **Kolmogorov-Smirnov test (K-S test)** for comparing the PRE curves with the remaining three curves in other conditions. All the statistics D listed here have p-values $p < 10^{-6}$ unless reported otherwise: $^{a}p = 0.0464$, $^{b}p = 0.592$ (n.s.). 6

S1 Dataset detailed figures

Figure S1 plots the distributions of tweet volumes for the hours preceding and following the one-hour window we analyzed. We show one instance for each category. The peaks for the debates correspond to the first hour of each debate, the peak for the RNC corresponds to Clint Eastwood's famous keynote directed at an empty chair at 21:00 EDT on August 30, the peak for the Benghazi attacks corresponds to 20:00 EDT on September 11 when the first American fatality was reported.

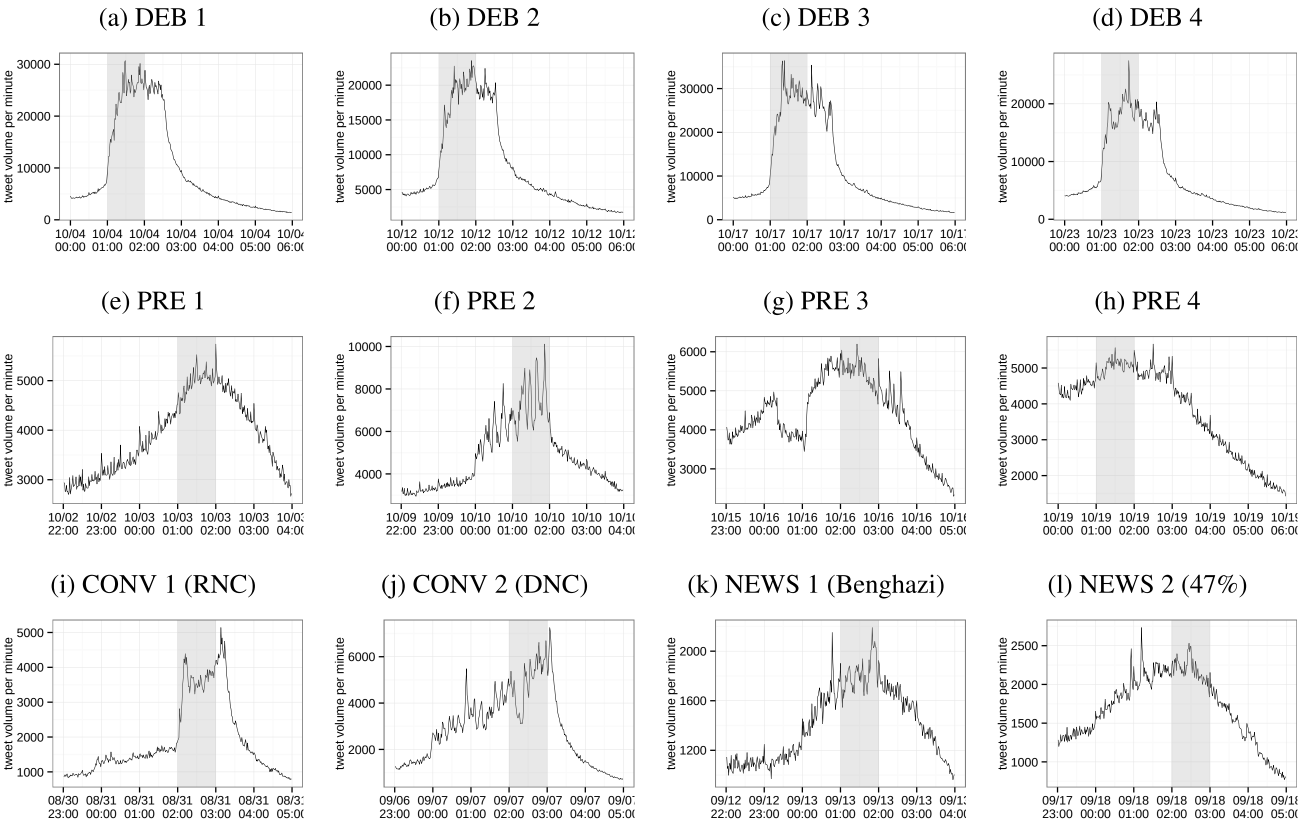

Figure S1: Tweet volume per minute. Number of tweets per minute in the 12 datasets. (a-d) The six hours during the four debate events ("DEB"). For other categories, we plot the six hour volume centering around the peak within the data range: (e-h) Normal period prior to the debate evenings ("PRE"). (i,j) National convention events including RNC and DNC ("CONV"). (k,l) Breaking political news events including Benghazi attack and Romney's 47-percent video ("NEWS").

S2 Features of communication

Tweets encode a variety of relational features such as hashtags, mentions, replies, and retweets. Using these features, we construct networks to measure the distributions of users', hashtags', and tweets' centralities.

- **Hashtags** (#) – This user-to-hashtag network models when user Alice mentions hashtag "#foo" in a tweet. Alice's hashtag out-degree reflects the number of hashtags she has referenced and the hashtag in-degree of "#foo" reflects the number of users referencing it.
- **Mentions** (@) – This user-to-user network models when user Alice addresses user Bob anywhere in her tweet. This notifies Bob and lets anyone following her see the tweet. Alice's mention out-degree reflects the number of unique users she has mentioned in her tweets and Bob's mention in-degree reflects the number of unique users who have mentioned him in their tweets.
- **Replies** – This user-to-user network models when user Alice addresses user Bob as the start of her tweet. This notifies Bob and only lets the users following both of them see the tweet. Alice's reply out-degree reflects the number of unique users she has replied to in her tweets and Bob's reply in-degree reflects the number of unique users who have replied to him in their tweets.

- **Retweets (RT)** – This user-to-tweet network models when user Alice repeats a tweet from user Bob. Alice's retweet out-degree reflects the number of tweets she has re-tweeted and Bob's tweet in-degree reflects the number of users who have retweeted that tweet. The in-degrees of all of Bob's tweets can be summed to make a **Retweet user in-degree (RT user)** reflecting the number of times all of Bob's tweets have been retweeted by all other users.

S3 Patterns of other activity networks

S3.1 Changes in communication (mention)

Figure S2 plots the changes in the mention ratio for the twelve observations grouped by event type (typical, news event, national convention, and debate). Similar to the reply ratio (shown in the **Main** text), the ratio of tweets that include any mentions of users in the tweet (Fig. S2) declines substantially during media events like the debates.

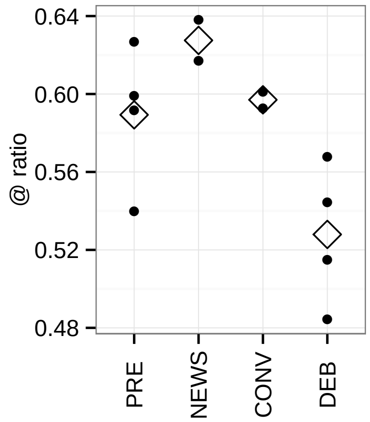

Figure S2: Changes in communication volume. Diamond shapes indicate the mean value of each category. This figure shows the ratio of tweets mentioning a user to the total tweets at the peak hour.

S3.2 Changes in distribution

Figure S3 plots the in- and out-degree Lorenz curves for the networks of hashtags and mentions. The top users are responsible for most hashtag references (Fig. S3(c)), and mentioning other users (Fig. S3(d)). Along with Fig. 2(c,d) in the **Main** text, the out-degrees for all four types of activity networks show significant similarities across the four event types and comparatively high levels of concentrated activity. These findings together suggest the concentration of users' attention to content in the networks is very similar regardless of media events.

The top 20% of hashtags make up more than 50% of all observed hashtags (Fig. S3(a)), with this concentration being more exaggerated around the national conventions and more relaxed around the news events. This reflects the dominance of a official hashtags like "#nbcpolitics" during the conventions and the presence of many unrelated but popular hashtags like "#moviesyoucantdislike" during the unscheduled news events. The convention and debate media events also drove increased concentration of mention (Fig. S3(b)) and

reply activity (in **Main** text Fig. 2(b)) around top users as compared to pre-events and news events. This is suggestive of many users directing their tweets towards elite users rather than each other.

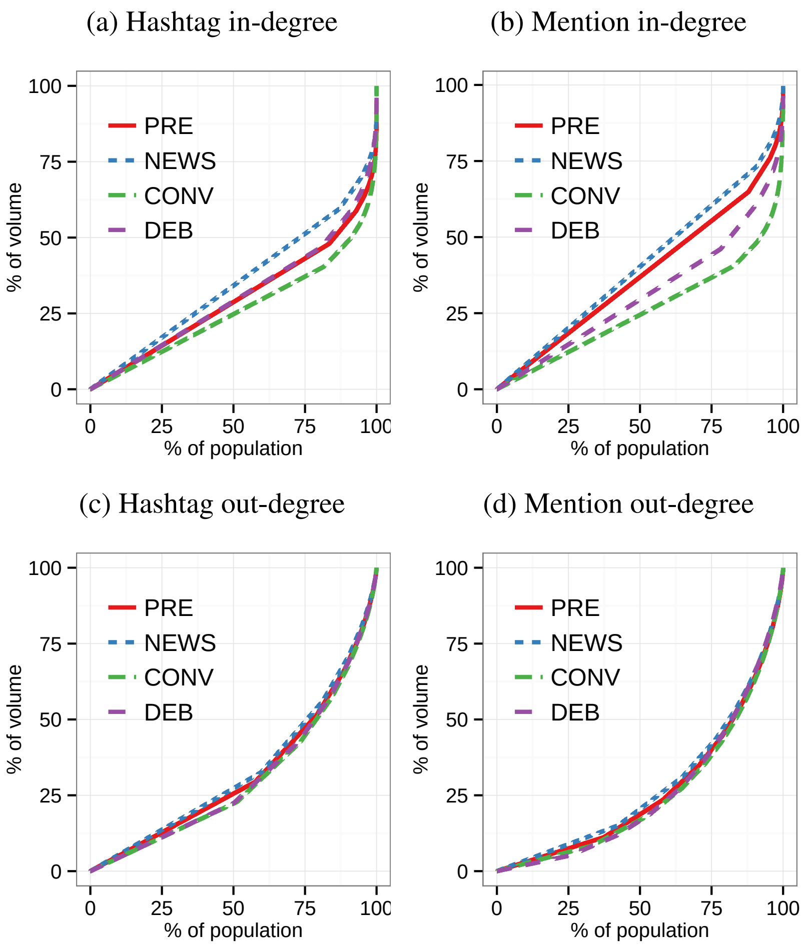

Figure S3: Lorentz curves for cumulative degree distributions of activity. Increasing equality converges toward diagonal line from the origin to the upper-right and increasing inequality converges toward a hyperbola rising to 100% of volume at the 100th percentile.

S3.3 Changes in concentration

Figure S4 shows the in- and out-degree statistics of user-to-hashtag network and user-to-user mention network. In the user-to-hashtag network (Fig. S4(a,c)), all twelve events show moderate amounts of centralization and low levels of connectivity. Users do not use many unique hashtags on average (Fig. S4(a)) nor are there more hashtags in circulation on average (Fig. S4(c)) during the media events compared to other types of events. There is more centralization among the hashtags than users, reflecting the dominance of official hashtags about events, but the differences between types of events are not major.

In the user-to-user mention network, media events have a greater tendency for a few users to receive many mentions from other users than other types of events (Fig. S4(b)) but most users still receive fewer than two mentions during the peak hour for all types of events. A similar pattern is found in the user-to-

user reply network (in **Main** text Fig. 3(a)), both of which can be interpreted as changes at the level of the system concentrating activity without corresponding changes at the level of the users increasing connectivity. Returning to the mention network, the mention out-degree spaces show that users mention between three and five other users on average over the course of the peak hours for all events (Fig. S4(d)) but much of this mentioning behavior in concentrated in a few, very "chatty" users communicating with very many other users. The reply network's out-degree (in **Main** text Fig 3(d)) has half the average connectivity of the mention network's out-degree, indicating users reply to only two other users during peaks across and this effect holds across all types of events.

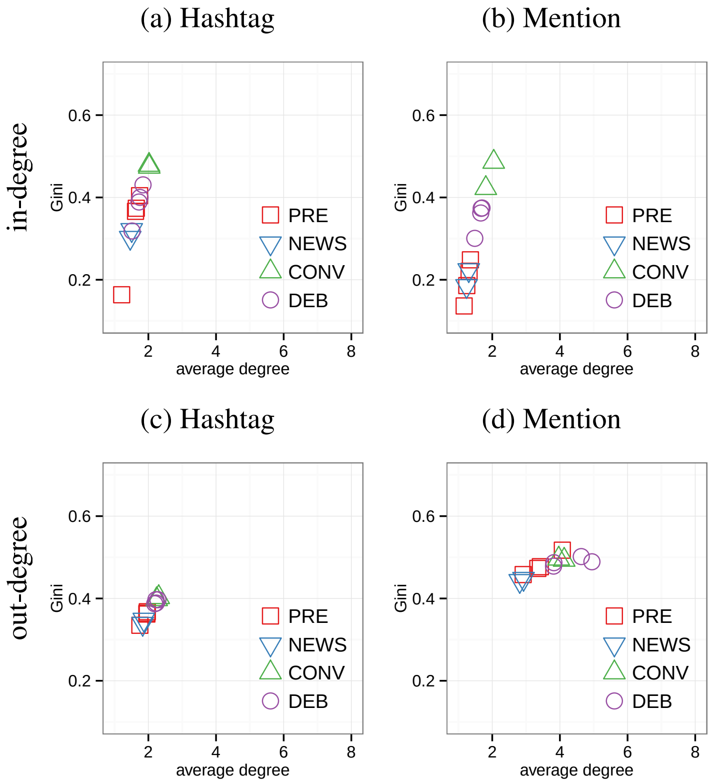

Figure S4: Connectivity-concentration state spaces. For each of the twelve observed events, the Gini coefficient for the network's degree distribution is plotted on the *y*-axis and the average degree of the network is plotted on the *x*-axis.

S4 Statistics of changes in distribution

To test whether the concentration of activity differed significantly from the pre-debate event baselines, we measured the deviation of each event type's Lorenz curve from the pre-debate baseline events using a two-sample Kolmogorov-Smirnov (K-S) test [1]. The K-S statistics in Table S1 confirm the Lorenz curves for networks of mentions, replies and retweets are significantly different during the news events, national conventions, and debates. The statistics for the differences between the out-degrees are generally much larger than the statistics for the in-degrees, reflecting the larger differences between these curves discussed above. Taken together, users reproduce similar *behaviors* of focusing their activity on certain tweets, users, and hashtags across all types of events even though the tweets, users, and hashtags that are the *focus* of this

attention becomes more concentrated during media events.

The K-S test is a nonparametric test for difference between two continuous, one-dimensional distributions and is given by:

$$D = sup_x|F_1(x) - F_2(x)|,$$

where in our case, F_1 and F_2 are two Lorenz curve functions.

Table S1: Kolmogorov-Smirnov test (K-S test) for comparing the PRE curves with the remaining three curves in other conditions. All the statistics D listed here have p-values $p < 10^{-6}$ unless reported otherwise: $^a p = 0.0464$, $^b p = 0.592$ (n.s.).

		Hashtag	Mention	Reply	RT user
in-deg.	NEWS	0.133	0.074	0.040	0.108
	CONV	0.113	0.280	0.145	0.339
	DEB	0.019^a	0.158	0.162	0.274
out-deg.	NEWS	0.062	0.060	0.067	0.095
	CONV	0.066	0.052	0.011^b	0.091
	DEB	0.037	0.083	0.091	0.125

References

[1] Gail MH, Gastwirth JL (1978) A scale-free goodness-of-fit test for the exponential distribution based on the gini statistic. Journal of the Royal Statistical Society Series B (Methodological) : 350357.
